# Supplementary material for: Ketogenic diet reduces early mortality following traumatic brain injury in Drosophila via the PPARγ ortholog Eip75B
Source: PLoS One. 2021 Oct 26;16(10):e0258873. doi: 10.1371/journal.pone.0258873 (PMC8547619; doi:10.1371/journal.pone.0258873)
Supplement: Attachment — Submitted filename: Response to Reviewers.docx [file pone.0258873.s002.docx]

Laboratory of Genetics

College of Agricultural and Life Sciences

School of Medicine and Public Health

425

Henry Mall, Madison, WI 53706

**http://www.genetics.wisc.edu**

(608) 262-1069

🞟

(608) 262-2976 Fax

**Responses to reviewer’s criticisms (in bold font)**

Reviewer #1: This manuscript has been improved by the revisions suggested by the Reviewers.

One minor point is to make clear along the y-axes that MI24 is a percentage. It is unlabelled and not clarified in the legends.
Also in the text on page on page 4, "varies from 7 to 58" should be "7% to 58% among 179...."

**We appreciate the reviewer’s suggestion. To increase clarity, we added the following sentence to the legends for Figures 1, 2, and 4. “The MI_24_ represents the percent mortality of injured flies minus the percent mortality of uninjured flies 24 h following TBI.”**

**Adding percent or % to the y-axis label is problematic because the MI_24_ is defined as a percent. So, MI_24_ percent and MI_24_% are redundant and percent mortality and % mortality are incorrect. Furthermore, I would like the graphs to be consistent with our prior publications. We have published graphs in nine papers with y-axes labeled MI_24_, and other labs have also used this nomenclature.**

Reviewer #2: It is surprising that the authors were unwilling to accommodate minor changes to the text that would have helped emphasize the importance of this work to a broader audience. While my recommendation is to accept the paper based on its technical soundness and its improvement from the review process, I would encourage the authors to be more flexible in the future.

**We thank the reviewer for the suggestion.**
